# Revumenib-Induced QTc Prolongation Leading to Multiple Episodes of Torsades de Pointes and Ventricular Arrhythmias

**DOI:** 10.31486/toj.25.0100

**Published:** 2026

**Authors:** Casey L. Keller, Kristina C. Hermanson, John C. Tawwater, Ryan M. Schuller, Diane J. St. Pierre, Harris V. Naina, Saeed K. Alzghari

**Affiliations:** ^1^ Department of Pharmacy, Texas Health Harris Methodist, Fort Worth, TX; ^2^ Department of Hematology, Texas Oncology, Fort Worth, TX

**Keywords:** *Arrhythmias–cardiac*, *drug-related side effects and adverse reactions*, *histone-lysine N-methyltransferase*, *humans*, *leukemia–myeloid–acute*

## Abstract

**Background:**

Revumenib is the only oral menin inhibitor approved for the treatment of relapsed/refractory acute leukemia with menin–lysine methyltransferase 2A (KMT2A) rearrangement in adults and pediatric patients aged >1 year. Revumenib was granted US Food and Drug Administration approval under the accelerated approval pathway, receiving both breakthrough therapy and orphan drug designations because of its potential to address a significant unmet need for patients with relapsed/refractory acute leukemia with a KMT2A translocation. While revumenib has no absolute contraindications, the package insert carries warnings, including the risk of corrected QT (QTc) prolongation.

**Case Report:**

A 76-year-old male with relapsed/refractory acute myeloid leukemia received revumenib after exhausting all other treatment options because of failure or intolerance. During treatment, the patient experienced multiple episodes of torsades de pointes and ventricular arrhythmias. Given the gravity of the patient's disease and the lack of alternative therapeutic options beyond hospice, a multidisciplinary team implemented aggressive measures to mitigate arrhythmic risk: placement of a permanent pacemaker following the initial torsades de pointes episode, meticulous electrolyte management, medication adjustments to limit QTc prolongation and drug-drug interactions, and use of a wearable cardioverter defibrillator. Despite a subsequent episode of torsades de pointes, the patient continued revumenib for an additional 29 days without further arrhythmias until discontinuation because of worsening acute myeloid leukemia shown by bone marrow biopsy.

**Conclusion:**

Although current guidance recommends discontinuation of revumenib in the setting of life-threatening arrhythmias, this case highlights the importance of individualized risk-benefit assessment and supportive strategies when treatment is pursued as a last resort.

## INTRODUCTION

Acute leukemia is a malignancy characterized by genetic alterations in hematopoietic cells, resulting in impaired differentiation and uncontrolled cellular proliferation.^1^ The development of relapsed/refractory leukemia presents a significant clinical challenge, particularly in older adults because of diminished treatment tolerance and increased incidence of adverse genomic features. Despite therapeutic advancements, outcomes in relapsed/refractory acute leukemias remain poor, with no established standard of care and 5-year overall survival rates of approximately 50% in acute lymphoblastic leukemia and 17% in acute myeloid leukemia.^2^ Among the genetic anomalies implicated in acute leukemia, rearrangements of the lysine methyltransferase 2A (KMT2A) gene, formerly known as MLL (mixed-lineage leukemia), are found in up to 10% of cases.^3^ These KMT2A rearrangements are associated with resistance to conventional therapies and a particularly poor prognosis, with <10% of adult patients achieving complete remission following 3 or more lines of therapy.^4^

Revumenib (SNDX-5613; Revuforj) is a first-in-class oral oncolytic agent that inhibits the interaction between the KMT2A protein and menin by binding to the menin-binding motif, thereby preventing formation of the menin-KMT2A complex.^5^ The accelerated approval by the US Food and Drug Administration was based on the results of the AUGMENT-101 trial that showed revumenib monotherapy was well tolerated and offered higher remission rates compared to historic controls in patients with high-risk, relapsed/refractory KMT2A-rearranged acute leukemia.^6,7^ The AUGMENT-101 trial reported an overall response rate of 63.2% and allowed a subset of patients to proceed to an allogeneic hematopoietic stem cell transplant, a potentially curative therapy.^7^ Because revumenib is primarily metabolized by cytochrome P450 3A4 (CYP3A4), the pharmacokinetics are modulated by coadministration of CYP3A4 inhibitors or inducers, requiring careful monitoring of concurrent medications and dose reductions when strong CYP3A4 inhibitors are used.^8^

Among reported adverse effects, corrected QT (QTc) prolongation has emerged as the primary dose-limiting toxicity of revumenib, although the underlying mechanism is unknown.^2,9^ The package insert for revumenib recommends an electrocardiogram (ECG) prior to initiation of therapy, weekly ECGs for the first 4 weeks of treatment, and then monthly or as clinically indicated.^8^ Treatment interruption is recommended for a QTc interval (Fridericia correction) >480 ms, and permanent treatment discontinuation is recommended for ventricular arrhythmia or QTc prolongation with life-threatening arrhythmia.^8^ Notably, QTc prolongation was reversible in the AUGMENT-101 study with management strategies including electrolyte repletion, withholding revumenib for QTc >481 ms, and dose reduction for persistent prolongation.^9^

We report a case of revumenib-induced QTc prolongation resulting in life-threatening ventricular arrhythmias in a patient with relapsed/refractory acute myeloid leukemia and illustrate the complexities of toxicity mitigation to preserve this critical last-line therapy.

## CASE REPORT

A 76-year-old male presented to the emergency department from an outside hospital with complaints of shortness of breath and back pain. His medical history included hypothyroidism, hypertension, hyperlipidemia, and metastatic prostate cancer (status post radiation and leuprolide treatment for 10 years that was discontinued because of remission). His surgical history included cholecystectomy, appendectomy, tonsillectomy, hernia repair, prostatectomy, colonoscopy, and laser-assisted in situ keratomileusis and cataract surgery in the right eye. He had no relevant family history.

The patient reported severe weakness, anorexia, cough, nausea, low-grade fevers, a pruritic rash on the abdomen, and 1 episode of blood in his stool during the prior 2 to 3 weeks. His initial oxygen saturation was 82% on room air, white blood cell count was 1.08 k/μL (reference range, 4-11 k/μL), hemoglobin was 7 g/dL (reference range, 13-17 g/dL), hematocrit was 19.7% (reference range, 38%-51%), and platelets were 32 k/μL (reference range, 140-416 k/μL). Oncology was consulted, and the patient was admitted to the hospital for transfusion support. Peripheral blood smear showed 32% blasts, and a bone marrow biopsy was performed on day 2. He was transferred to the progressive care unit the same day for hi-flow oxygen requirements because of pulmonary infiltrates and to initiate treatment for pneumonia with cefepime 2,000 mg intravenously (IV) once, followed by meropenem 2,000 mg IV every 8 hours for 15 days.

On day 6, the patient was transferred to a large tertiary care center that performs a high volume of induction therapy in the treatment of acute leukemia. The bone marrow biopsy performed at the first institution confirmed acute myeloid leukemia, with the marrow showing approximately 40% blasts with dyserythropoiesis and the peripheral blood demonstrating pancytopenia with dysplastic neutrophils. The pathology report endorsed the classification of therapy-related acute myeloid leukemia because of the patient's history of receiving large field radiation. The patient then developed an acute kidney injury requiring intermittent hemodialysis, and treatment was deferred until he was clinically stable with improved oxygenation.

Chemotherapy began on day 14 with single agent decitabine 20 mg/m^2^ IV for 10 days, with the addition of other agents dependent on patient tolerance. Next-generation sequencing showed complex cytogenetics with deletion of 5q and 7q, KMT2A amplification, and p53 mutation. On day 19, venetoclax was initiated at a reduced dose of 100 mg orally daily for 28 days because of concurrent drug-drug interaction with voriconazole that had been initiated on day 6. At this time, the patient had achieved renal recovery and did not require further dialysis.

On day 34, a second bone marrow biopsy revealed approximately 30% blasts, and monotherapy with IV cytarabine 20 mg twice daily was started on day 37 for a planned duration of 5 days. The patient remained profoundly neutropenic with an absolute neutrophil count of zero (reference range, 1.5-7.9 k/μL) on opportunistic infection prophylaxis consisting of levofloxacin 500 mg orally every 48 hours for 21 days, micafungin 100 mg IV daily for 77 days, and valacyclovir 500 mg orally daily for 134 days.

Because of the KMT2A mutation, revumenib was initiated on day 40 at a dose of 270 mg orally twice daily. ECG showed a baseline QTc of 455 ms, and ECG was repeated daily thereafter. Potassium and magnesium were closely followed and optimized according to an institution electrolyte replacement protocol with a potassium goal of 3.9 mmol/L and magnesium goal of 1.8 mEq/L. Levofloxacin was changed to amoxicillin-clavulanate 875/125 mg orally twice daily on day 41 to minimize QTc prolongation. On day 43, the patient had a fever of 100.5 ºF, and amoxicillin-clavulanate was changed to cefepime 2,000 mg IV every 8 hours plus vancomycin dosed per patient-specific pharmacokinetics for a goal area under the curve (AUC) of 400 to 600 mg•h/L.

On day 47, QTc was 507 ms, and revumenib was held for a day and resumed after resolution of prolongation. Shortly after, cefepime was de-escalated to levofloxacin at a dose of 500 mg orally daily for opportunistic infection prophylaxis. QTc became prolonged once more at 563 ms, and revumenib was again held for a day, as QTc normalized after switching antibacterial prophylaxis to cefpodoxime 200 mg orally twice daily.

On day 64, a morning ECG demonstrated sinus rhythm with first-degree atrioventricular block and occasional premature ventricular contractions with a QTc of 487 ms. That evening, the patient had 2 witnessed episodes of seizure-like activity and was noted to have polymorphic ventricular tachycardia. Magnesium 2 g IV and amiodarone 150 mg IV were administered, and subsequent ECG showed a junctional rhythm of 52 beats per minute and QTc of 478 ms (Tisdale Risk Score of 1). Recurrent runs of ventricular tachycardia occurred, and a second dose of amiodarone was administered. Repeat ECG showed sinus bradycardia with a rate of 47 beats per minute and QTc of 494 ms. The patient was transferred to the progressive care unit and subsequently developed polymorphic ventricular tachycardia in the context of sinus arrest with QTc prolongation consistent with torsades de pointes. Magnesium 4 g IV was administered, and the patient was started on isoproterenol 2 μg/min IV titrated to a heart rate goal of >100 beats per minute to shorten the QT interval. Revumenib was held, and no further amiodarone was administered to avoid further bradycardia and QTc prolongation. The patient was transferred to the cardiac intensive care unit (ICU) for closer monitoring.

A bone marrow biopsy performed on day 64 showed an increase in blasts to 55.7%. Five days later, treatment with azacitidine 75 mg/m^2^ IV and venetoclax 400 mg orally was initiated, both for 7 days. On day 70, Cardiology placed a permanent pacemaker with a goal heart rate of 80 beats per minute to shorten the intrinsic QT interval and avoid torsades de pointes while the patient was on revumenib; after the procedure, the patient was transferred out of the cardiac ICU. The following day, the patient reported severe nausea with azacitidine and did not want further treatment with this agent. He was switched to decitabine 20 mg/m^2^ IV for 3 doses, for a total of 5 days of therapy with a hypomethylating agent. The day after, revumenib was restarted at 270 mg twice daily with continued telemetry monitoring. On the third day of this course, nursing held the morning dose of revumenib because of prolonged QTc; however, because of the presence of a pacemaker, the QTc was not considered to be a reliable predictor of subsequent development of torsades de pointes, and pharmacy provided education to nursing to restart revumenib on day 74 irrespective of QTc that continued for 18 days.

On the evening of day 92, the patient complained of headache and dizziness, and he became briefly unresponsive. He was hypotensive and bradycardic at 48 beats per minute. ECG and head computed tomography (CT) were ordered, and the pacemaker was interrogated. Later that night, the patient had a 5-beat run of ventricular tachycardia and subsequent torsades de pointes (Tisdale Risk Score of 1). Magnesium 2 g IV was administered, and the patient was again transferred to the cardiac ICU for a higher level of care. The following morning, the patient's rhythm changed to ventricular fibrillation/torsades de pointes, and cardiopulmonary resuscitation was started. Epinephrine 1 mg IV was administered, with return of spontaneous circulation achieved after 2 minutes without the need for defibrillation. An additional dose of magnesium 2 g IV was given. Revumenib was once again held.

CT angiography showed no significant coronary disease, suggesting that ischemia-driven polymorphic ventricular tachycardia was not contributing to the arrhythmias. Cardiology planned to upgrade the dual-chamber pacemaker to a dual-chamber implantable cardioverter defibrillator once the patient's hematologic derangements and thrombocytopenia improved. The patient continued to be paced at 80 beats per minute during this time, and because of profound thrombocytopenia (platelet count of 10 k/μL [reference range, 140-416 k/μL]), treatment was supplemented with a wearable cardioverter defibrillator.

The patient became febrile with a temperature of 102.8 ºF, and cefpodoxime was changed to cefepime 2,000 mg IV every 8 hours. On day 96, the patient was transferred out of the cardiac ICU, and bone marrow biopsy revealed 40% to 50% blasts. The option of hospice was discussed, but the patient and his family declined. On day 103, treatment began with cladribine 5 mg/m^2^ IV for 5 days and venetoclax 400 mg for 28 days. Revumenib was resumed at the same dose as previously given. At this time, sulfamethoxazole/trimethoprim 800/160 mg orally 3 times weekly was started for *Pneumocystis jirovecii* pneumonia prophylaxis. Upon initiation of this cycle, the patient had sporadic premature ventricular contractions that resolved spontaneously.

On day 106, antifungal prophylaxis was changed from micafungin 100 mg IV daily to isavuconazonium 372 mg orally daily because of QT shortening effects, necessitating a venetoclax dose decrease to 200 mg because of drug-drug interaction. Additionally, sulfamethoxazole/trimethoprim was changed to inhaled pentamidine 300 mg every 4 weeks for *P jirovecii* pneumonia prophylaxis as the patient's potassium continued to trend upwards (5.1 mmol/L). Electrolytes were optimized, and the patient received daily magnesium IV infusions. On day 117, a peripheral blood smear revealed no blasts.

On day 119, the patient developed fevers with a temperature of 101.6 ºF. Cefpodoxime was discontinued; meropenem 2,000 mg IV every 8 hours and vancomycin dosed per patient-specific pharmacokinetics for a goal AUC of 400 to 600 mg•h/L were started for treatment of neutropenic fever. On day 121, blood cultures grew *Serratia*, and meropenem was switched to cefepime 2,000 mg IV every 8 hours.

On day 131, bone marrow biopsy revealed a worsening of acute myeloid leukemia: blasts of 80% with grade 2 fibrosis. The following day, revumenib was discontinued after a total of 29 days. Further treatment options were explored elsewhere; however, no further treatment was recommended. The patient declined hospice treatment and was discharged home on supportive care.

## DISCUSSION

In our patient, a Naranjo score of 9 supported definite causality between revumenib exposure and QTc prolongation leading to torsades de pointes (Table).^10^ The Naranjo score is calculated based on the responses to 10 questions answered yes, no, or do not know. Different point values (–1, 0, +1, or +2) are assigned to each answer. An adverse drug reaction is considered definite if the score is 9 to 13, probable if the score is 5 to 8, possible if the score is 1 to 4, and doubtful if the score is 0 to –4.^10^

**Table.** Naranjo Adverse Drug Reaction Probability Scale Evaluation of the QTc Prolongation in our Patient After Administration of Revumenib

Question	Yes	No	Do Not Know	Score for Our Patient
1. Are there previous conclusive reports on this reaction?	+1	0	0	+1^a^
2. Did the adverse event appear after the suspected drug was administered?	+2	–1	0	+2^a^
3. Did the adverse event improve when the drug was discontinued or a specific antagonist was administered?	+1	0	0	+1^a^
4. Did the adverse event reappear when the drug was readministered?	+2	–1	0	+2^a^
5. Are there alternative causes that could on their own have caused the reaction?	–1	+2	0	+2^b^
6. Did the reaction reappear when a placebo was given?	–1	+1	0	0^c^
7. Was the drug detected in blood or other fluids in concentrations known to be toxic?	+1	0	0	0^c^
8. Was the reaction more severe when the dose was increased or less severe when the dose was decreased?	+1	0	0	0^b^
9. Did the patient have a similar reaction to the same or similar drugs in any previous exposure?	+1	0	0	0^b^
10. Was the adverse event confirmed by any objective evidence?	+1	0	0	+1^a^
**Total score for our patient**				**9**

^a^The response to these questions was “yes” for our patient.

^b^The response to these questions was “no” for our patient.

^c^The response to these questions was “do not know” for our patient.

Note: The Naranjo score is calculated based on the yes, no, or do not know responses to these 10 questions, with different point values (–1, 0, +1, and +2) assigned to each response. An adverse drug reaction is considered definite if the score is 9 to 13, probable if the score is 5 to 8, possible if the score is 1 to 4, and doubtful if the score is 0 to –4.^10^

Drug-induced QT prolongation is a well-documented adverse effect. This abnormality predisposes patients to developing torsades de pointes, a malignant form of polymorphic ventricular tachycardia that may degenerate into ventricular fibrillation or sudden cardiac death if sustained.^11^ Timely recognition of QTc prolongation is therefore critical, as interventions such as withdrawal of the offending agent, correction of electrolyte abnormalities, and intensified cardiac monitoring can prevent escalation to life-threatening arrhythmias.^12,13^ Each 10 ms increase in QTc has been associated with a 5% increase in the risk of developing ventricular arrhythmias.^12^ The American Heart Association and the American College of Cardiology Foundation have established a QTc interval threshold of 470 ms for men and 480 ms for women as the upper limit of normal, with QTc values >500 ms considered abnormal for both sexes.^14^

The risk of developing torsades de pointes is multifactorial and influenced by both patient-specific and pharmacologic factors. Risk factors include female sex, advanced age, bradycardia, hypokalemia, hypomagnesemia, structural heart disease, and concomitant use of multiple QT-prolonging medications.^12^ The multifactorial risk of developing torsades de pointes complicates the therapeutic picture, particularly now that oncology is increasingly embracing novel targeted agents whose potential to cause arrhythmias is not fully understood.

Historically, patients with relapsed/refractory acute myeloid leukemia have had dismal outcomes and severely restricted treatment options, so revumenib represents a meaningful therapeutic advancement, offering the potential for remission where few alternatives exist. Nevertheless, revumenib is an example of the dilemma associated with accelerated approval pathways for drugs whose efficacy may be promising but their long-term safety profiles and rare but severe adverse drug reactions are incompletely characterized. Experience reinforces this concern. A 2002 study published in the *Journal of the American Medical Association* showed that between 1975 and 1999, 10.2% of newly approved drugs either acquired a boxed warning or were withdrawn from the market.^15^ Probability models suggest a 20% likelihood of such outcomes within 25 years.

Across multiple clinical trials, including SAVE, Beat AML, AUGMENT-101, and AUGMENT-102, revumenib demonstrated consistent remission rates in both adult and pediatric patients, positioning it as the frontrunner in the menin inhibitor class.^7,16-18^ However, QTc prolongation has emerged consistently as a notable toxicity. Reported incidences of all-grade QTc prolongation ranged from 15% to 58% across trials, with 0% to 13.8% of patients experiencing grade 3 QTc prolonging events (severe or medically significant but not immediately life threatening); no grade 4 or grade 5 prolongations have yet been described, and, in the trials, QTc prolongation resolved with drug interruptions or dose adjustments.^7,16-18^ The pathophysiology of QTc prolongation caused by revumenib remains unclear. Trial data are constrained by small patient numbers and ongoing follow-up, limiting the ability to detect rare but serious arrhythmias such as torsades de pointes.^19^

Further complicating patient management are metabolic interactions with CYP3A4, the principal enzyme responsible for revumenib elimination. Coadministration with strong inhibitors increases systemic exposure 2- to 2.5-fold, elevating the risk of arrhythmia and necessitating dose reductions. Conversely, concomitant use with CYP3A4 inducers decreases revumenib exposure but increases levels of its M1 metabolite, which may itself contribute to QT prolongation.^8^ Recognizing these interactions is therefore crucial in preserving efficacy while minimizing toxicity, factors the pharmacists were actively monitoring in this patient's case.

Despite electrolyte management and the careful avoidance of concomitant QT-prolonging agents while on revumenib, our patient developed recurrent QTc prolongation, ventricular tachycardia, and ultimately torsades de pointes that necessitated intensive interventions. The cardiologist consulted identified sinus bradyarrhythmia and sinus arrest with transitions to polymorphic ventricular tachycardia. Isoproterenol was initiated to acutely manage bradycardia, reducing the prolongation of the QTc interval and risk of the R-on-T phenomenon that results in torsades de pointes. Subsequently, an electrophysiologist implanted a dual-chamber pacemaker with right atrial and right ventricular leads with a lower heart rate limit set to 80 beats per minute to mitigate bradycardia-related triggers. Increasing heart rate through the use of isoproterenol and electrical pacemakers is recommended in various international guidelines; however, breakthrough torsades de pointes has been reported to occur despite these therapies.^14,20^ Recurrent torsades de pointes despite electrical pacing at 80 beats per minute or more may occur as a result of device failure or as a side effect of the nonphysiological depolarization and repolarization sequence.^21,22^ Pacemakers set at rates of 50 to 60 beats per minute are commonly understood to not be particularly effective at preventing torsades de pointes, as intrinsic heart rates frequently exceed these values and the pause-dependent R-on-T phenomenon readily occurs.^23,24^ However, higher rates may not absolutely prevent pauses and early after-depolarizations when the innate tachycardia exceeds the intrinsic pacemaker rate or pauses are not readily suppressed by the pacemaker settings.^25^ Because these devices may not reliably prevent torsades de pointes, continued risk mitigation and monitoring should occur; however, the electrical sequelae of pacing devices produce artificially prolonged QRS and QTc intervals and make interpretation of torsades de pointes risk challenging. Numerous corrections for evaluation of paced QTc intervals have been proposed, but modifications of the QTc do not appear optimal for evaluation of proarrhythmic risk.^26^ Consideration of the JT interval or corrected JT interval during elevated heart rates may offer a novel alternative in that the JT interval more closely relates to the repolarization interval associated with proarrhythmia.^14,27^

## CONCLUSION

This case report emphasizes several key points. First, to our knowledge, this case is the first reported instance of recurrent torsades de pointes despite pacemaker placement in the context of revumenib therapy. Second, the case highlights the limitations of QTc interpretation in paced rhythms, creating diagnostic uncertainty in a setting where QT prolongation is a dose-limiting toxicity. Third, the case highlights the ethical and clinical complexity of balancing safety against therapeutic opportunity in patients with otherwise untreatable leukemias. While revumenib offers genuine potential for remission, evaluation of the drug is ongoing, and vigilance regarding life-threatening adverse drug reactions is essential. Shared decision-making, individualized care, and meticulous pharmacovigilance are indispensable when extending novel therapies to patients with limited therapeutic alternatives.

## Notes

*This article meets the Accreditation Council for Graduate Medical Education and the American Board of Medical Specialties Maintenance of Certification competencies for Patient Care and Medical Knowledge.*
